# Polymorphism and phase transformation in the dimethyl sulfoxide solvate of 2,3,5,6-tetra­fluoro-1,4-di­iodo­benzene

**DOI:** 10.1107/S2053229620005690

**Published:** 2020-04-30

**Authors:** Andrew D. Bond, Chris L. Truscott

**Affiliations:** a Department of Chemistry, University of Cambridge, Lensfield Road, Cambridge CB2 1EW, England

**Keywords:** halogen bonding, polymorphism, variable temperature, crystal structure, phase transformation, *PIXEL*

## Abstract

A new polymorph (form II) is reported for the 1:1 dimethyl sulfoxide solvate of 2,3,5,6-tetra­fluoro-1,4-di­iodo­benzene (TFDIB·DMSO or C_6_F_4_I_2_·C_2_H_6_SO). The structure is similar to that of a previously reported polymorph (form I) [Britton (2003 ▸). *Acta Cryst.* E**59**, o1332–o1333], containing layers of TFDIB mol­ecules with DMSO mol­ecules between, accepting I⋯O halogen bonds from two TFDIB mol­ecules. Re-examination of form I over the temperature range 300–120 K shows that it undergoes a phase transformation around 220 K, where the DMSO mol­ecules undergo re-orientation and become ordered. The unit cell expands by *ca* 0.5 Å along the *c* axis and contracts by *ca* 1.0 Å along the *a* axis, and the space-group symmetry is reduced from *Pnma* to *P*2_1_2_1_2_1_. Refinement of form I against data collected at 220 K captures the (average) structure of the crystal prior to the phase transformation, with the DMSO mol­ecules showing four distinct disorder com­ponents, corresponding to an overlay of the 297 and 120 K structures. Assessment of the inter­molecular inter­action energies using the *PIXEL* method indicates that the various orientations of the DMSO mol­ecules have very similar total inter­action energies with the molecules of the TFDIB framework. The phase transformation is driven by inter­actions between DMSO mol­ecules, whereby re-orientation at lower temperature yields significantly closer and more stabilizing inter­actions between neighbouring DMSO mol­ecules, which lock in an ordered arrangement along the shortened *a* axis.

## Abstract 2

The dimethyl sulfoxide solvate of 2,3,5,6-tetra­fluoro-1,4-di­iodo­benzene is polymorphic; one polymorph undergoes a phase transformation on cooling, associated with re-orientation of the dimethyl sulfoxide mol­ecules.

## Introduction   

The mol­ecule 2,3,5,6-tetra­fluoro-1,4-di­iodo­benzene (TFDIB) is a common halogen-bond donor (Metrangolo & Resnati, 2001 ▸; Cavallo *et al.*, 2016 ▸). The work described in this article originated from a cocrystal screening, where TFDIB was combined with a series of potential halogen-bond acceptors in dimethyl sulfoxide (DMSO) solution. Crystals of TFDIB·DMSO (see Scheme) were quite commonly obtained from these experiments, some of which were found to be different from a previously reported crystal structure at 297 K [Britton, 2003 ▸; Cambridge Structural Database (CSD; Groom *et al.*, 2016 ▸) refcode IKIFOX]. We refer to the previously reported structure (IKIFOX) as form I and the newly obtained polymorph as form II. The structures are similar and we suspected at first that form II might have arisen from a phase transformation on cooling of form I in the N_2_ cryostream during single-crystal data collection. We therefore obtained crystals of form I and measured them at various temperatures. We did not find any transformation of form I to form II, but instead observed re-orientation of the DMSO mol­ecules in form I to give a further new structure measured at 120 K. We describe herein the various crystal structures of TFDIB·DMSO and the application of dispersion-corrected DFT and *PIXEL* calculations (Gavezzotti, 2002 ▸, 2003 ▸, 2011 ▸) to examine the DMSO re-orientation on cooling of form I.
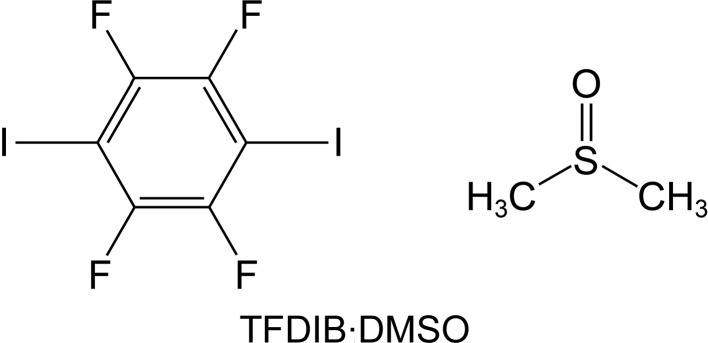


## Experimental   

### Synthesis and crystallization   

Crystals of forms I and II were produced during a sequence of attempted cocrystallization experiments. TFDIB and an anti­cipated coformer were dissolved in DMSO, and crystals were produced by vapour diffusion of water into the solution under ambient conditions. Form I (CSD refcode IKIFOX; Britton, 2003 ▸) was obtained frequently, while crystals of form II were obtained specifically from a 2:3 mixture of TFDIB and melamine (C_3_H_6_N_6_). The structure of form II was measured at 180 K, whereby the crystals were plunged directly from ambient conditions into a cold N_2_ stream. Similar treatment of form I resulted in cracking and the loss of single crystallinity. Analysis of form I was therefore made by placing the crystal initially into the N_2_ stream at room temperature, followed by slow cooling as described in §3.2.

### Refinement   

**Figure 1:**
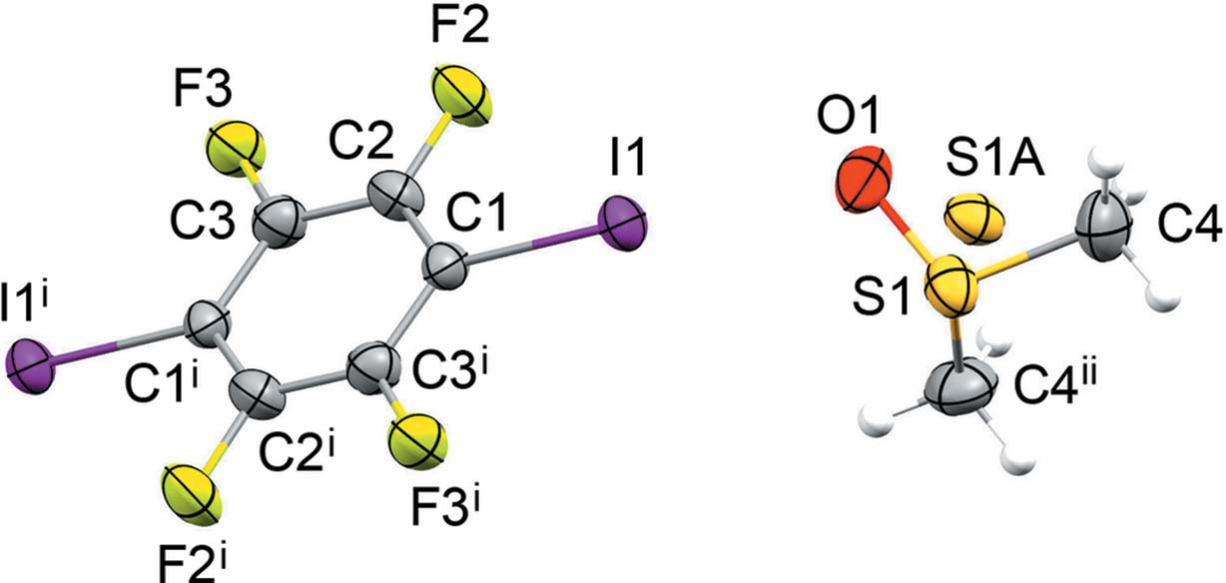
The mol­ecular structure of form II at 180 K, with displacement ellipsoids at the 50% probability level for non-H atoms. The site-occupancy factors for atoms S1 and S1*A* are 0.424 (5) and 0.576 (5), respectively. Only the major com­ponent is shown as connected. [Symmetry codes: (i) −*x* + 1, −*y* + 1, −*z*; (ii) *x*, −*y* + 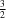, *z*.]

Crystal data, data collection and structure refinement details are summarized in Table 1 ▸. Determination of the structure of form II at 180 K was straightforward. The DMSO mol­ecule in the asymmetric unit is situated with its inter­nal mirror plane on the crystallographic mirror plane at *x*,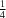,*z* in the space group *Pnma* (Fig. 1 ▸). The S atom is split into two atomic sites within the mirror plane, with refined site occupancies of 0.424 (5) and 0.576 (5).

**Figure 2:**
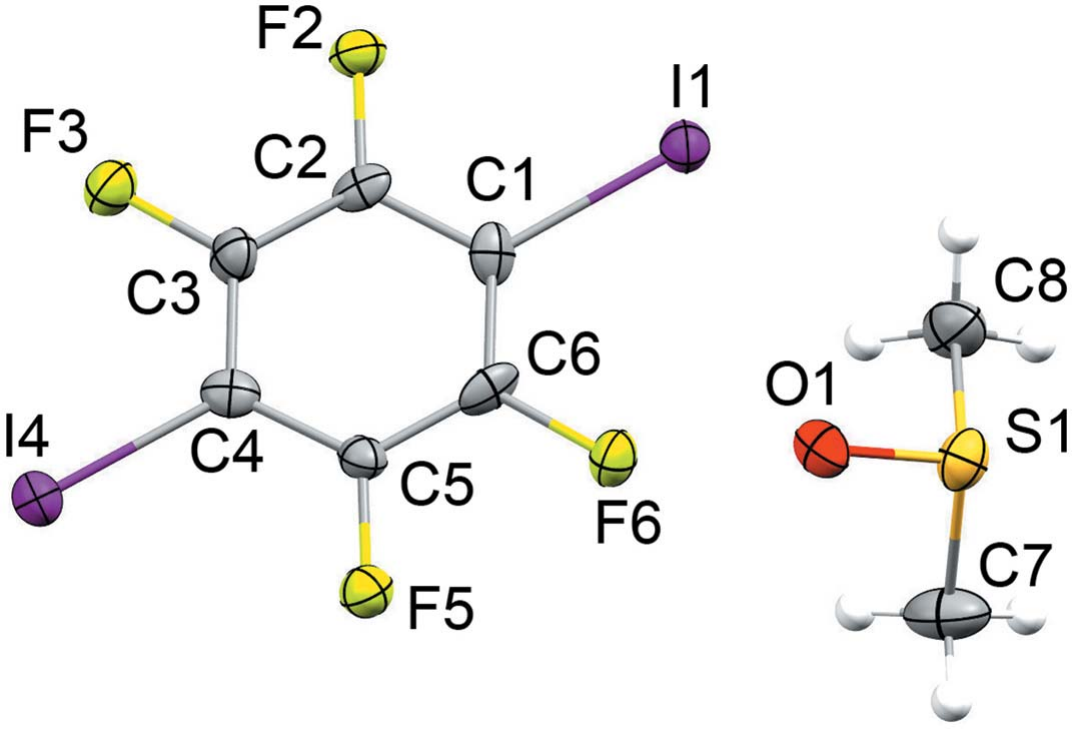
The mol­ecular structure of form I at 120 K, with displacement ellipsoids at the 50% probability level for non-H atoms.

For form I at 120 K, the space group is clearly *P*2_1_2_1_2_1_, with the DMSO mol­ecule ordered on a general equivalent position and with no significant residual electron density in the vicinity of the mol­ecule (Fig. 2 ▸). The structure was refined as an inversion twin with the Flack parameter converging to 0.19 (5). The applied unit-cell setting and origin (placing the 2_1_ screw axes at *x*,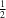,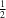; 0,*y*,0; 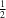,0,*z*) are nonstandard for the space group *P*2_1_2_1_2_1_ [Hall symbol: *P* 2*ac* 2*n*], but chosen to maintain the relationship with the form I structure at 297 K (IKIFOX) in its standard setting of *Pnma*.

**Figure 3:**
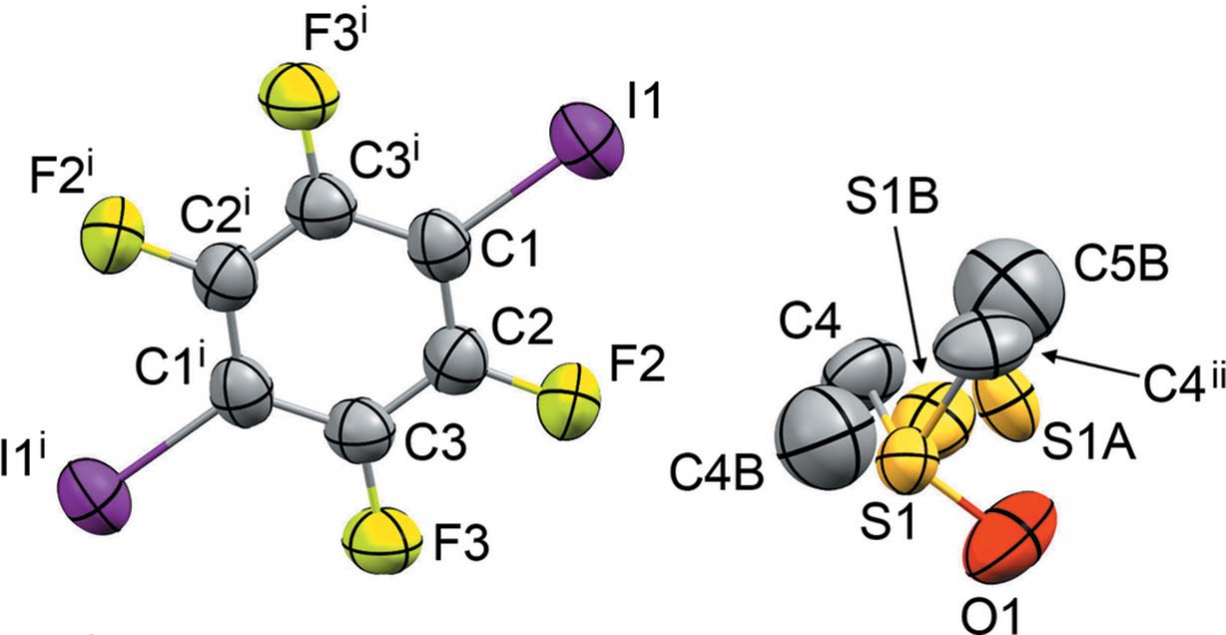
The mol­ecular structure of form I at 220 K, with displacement ellipsoids at the 50% probability level. H atoms have been omitted from the disordered DMSO mol­ecule for clarity, and only the major com­ponent is shown as connected. The site-occupancy factors for the DMSO com­ponents containing atom S1, S1*A* and S1*B* are 0.356 (3), 0.191 (3) and 0.226 (2), respectively. [Symmetry codes: (i) −*x* + 1, −*y* + 1, −*z* − 1; (ii) *x*, −*y* + 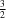, *z*.]

For the refinement of form I after cooling to 220 K, the DMSO mol­ecule was modelled in four orientations (Fig. 3 ▸). Two orientations are similar to those in form II, with the inter­nal mirror plane of the DMSO mol­ecule coincident with the mirror plane at *x*,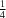,*z* in the space group *Pnma*, and with the S atom split into two atomic sites with refined site occupancies of 0.356 (3) and 0.191 (3). A further orientation is defined with the S atom out of the mirror plane with a refined site occupancy of 0.226 (2), giving two further orientations of the DMSO mol­ecule. The site occupancies of the three refined com­ponents were tightly restrained to sum to unity (using SUMP in *SHELXL*; Sheldrick, 2015*b* ▸) and restraints were applied to all S—O, S—C and C⋯C distances. All non-H atoms were refined with anisotropic displacement parameters, restrained to resemble isotropic behaviour (ISOR in *SHELXL*). An extinction coefficient was refined. In all struc­tures, the H atoms of the DMSO mol­ecule were placed in idealized positions, with *U*_iso_(H) = 1.5*U*_eq_(C). The methyl groups were not permitted to rotate around their local three­fold axes, since this prevented convergence of the refinement. The structure and refinement details are presented in Table 1 ▸.

### Computational details   

The crystal structures were energy-minimized with dispersion-corrected density functional theory (DFT-D) using the *CASTEP* module (Clark *et al.*, 2005 ▸) in *Materials Studio* (Accelrys, 2011 ▸). The PBE functional (Perdew *et al.*, 1996 ▸) was applied with a plane-wave cut-off energy of 520 eV, in combination with the Grimme semi-empirical dispersion correction (Grimme, 2006 ▸). The structures in the space group *Pnma* were reduced to the space group *P*2_1_2_1_2_1_ to allow the definition of com­plete mol­ecules, so all optimizations were carried out in *P*2_1_2_1_2_1_. The unit-cell parameters were constrained to the experimental values. For the disordered structures, models were built containing the various individual DMSO com­ponents and optimized separately. The DFT-D-optimized structures were used as input for the *PIXEL* module of the CSP package (Gavezzotti, 2002 ▸, 2003 ▸, 2011 ▸) to examine the energies of the pairwise inter­molecular inter­actions. The calculated inter­action energies are estimated to have accuracy within the range *ca* ±3 kJ mol^−1^.

## Results and discussion   

### Structure of form II   

**Figure 4:**
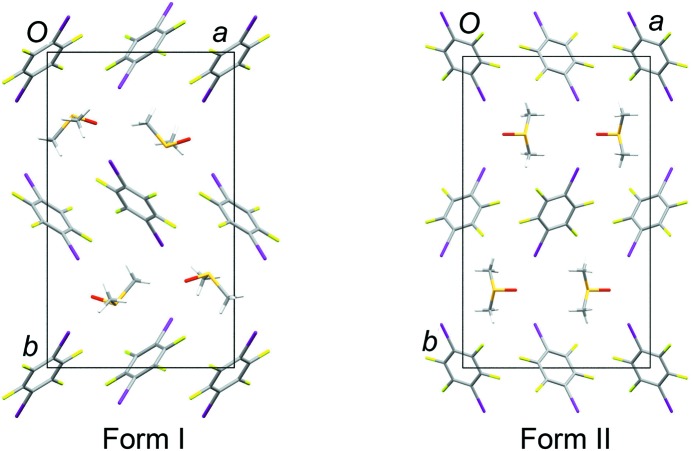
The form I and II structures, viewed along the *c* axis, showing layers of TFDIB mol­ecules in the (020) planes. The arrangement of TFDIB mol­ecules in form I is closely com­parable at 297 and 120 K (the 120 K structure is shown).

**Figure 5:**
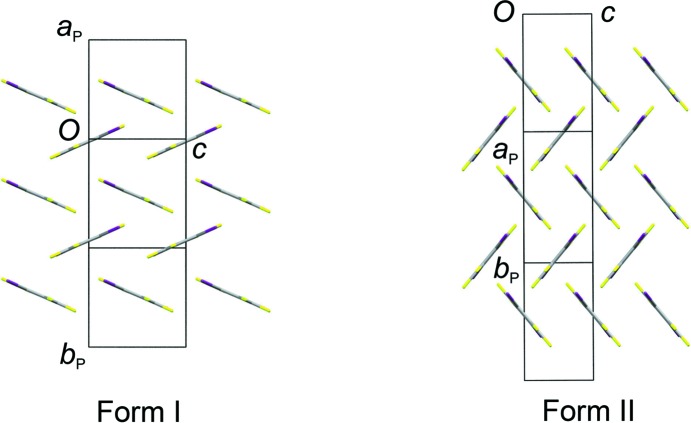
A single layer of TFDIB mol­ecules, looking side-on to the mol­ecules [projecting approximately onto the (110) planes]. Form I shows a smaller angle between the mol­ecular planes and has a longer *c* axis. The arrangement of TFDIB mol­ecules in form I is closely com­parable at both 297 and 120 K (the 120 K structure is shown).

Both form I and form II adopt structures with a layered arrangement of TFDIB mol­ecules in the (020) planes (Fig. 4 ▸). The DMSO mol­ecules occupy sites between these layers. The difference between forms I and II reveals some flexibility in the structure of the TFDIB layers within the crystalline state. Taking one layer and looking side-on to the mol­ecules [projecting onto the (110) planes], form II shows an approximately perpendicular arrangement of mol­ecules, while form I shows a smaller angle between the mol­ecular planes (Fig. 5 ▸). This difference is reflected in the unit-cell parameters (Table 1 ▸), particularly in the substanti­ally shorter *c* axis for form II. The sites occupied by the DMSO mol­ecules between the TFDIB layers in form II are substanti­ally similar to those in form I, as described in §3.2. The DMSO mol­ecules lie on the crystallographic mirror planes at *x*,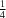,*z* and *x*,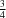,*z*, accepting I⋯O halogen bonds from two TFDIB mol­ecules either side of the mirror plane [I1⋯O1 = 2.847 (2) Å]. Disorder is present in the manner described for form I at 297 K (§3.2), with the DMSO mol­ecules adopting orientations **A** and **B** with refined site occupancies of 0.576 (5) and 0.424 (5), respectively.

### Temperature-dependent structure of form I   

**Figure 6:**
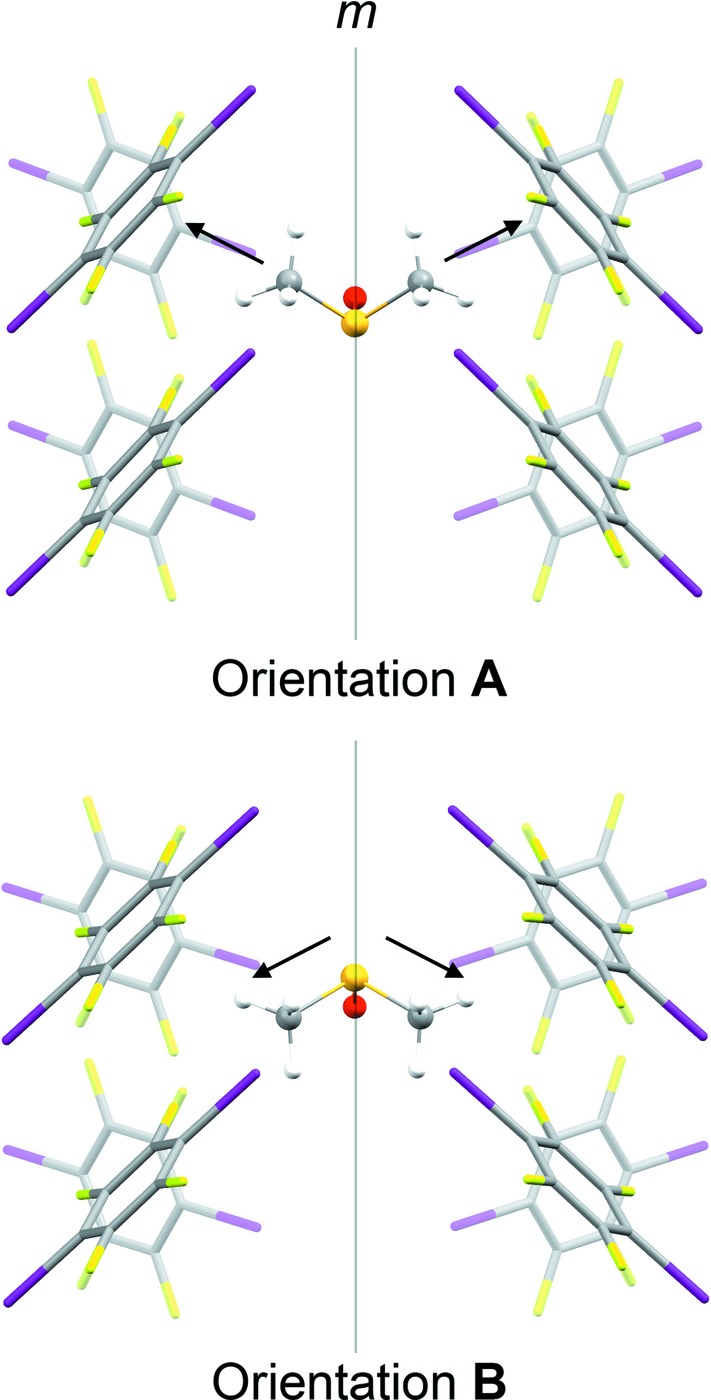
Two orientations (**A** and **B**) of the DMSO mol­ecule in the form I structure at 297 K (Britton, 2003 ▸). The mol­ecules occupy a site between eight TFDIB mol­ecules. The arrows indicate the directions of the S—CH_3_ bond vectors, *i.e.* perpendicular (**A**) or parallel (**B**) to the planes of the two TFDIB mol­ecules at the top in the front plane.

The previously-reported structure of form I at 297 K (Britton, 2003 ▸; CSD refcode IKIFOX) exhibits two orientations of the DMSO mol­ecules (labelled **A** and **B**), as illustrated in Fig. 6 ▸. We obtained an identical disorder model in our own refinements at 300 K (not reported). Each DMSO mol­ecule lies on a mirror plane in a pocket between eight TFDIB mol­ecules. The position of the O atom is approximately consistent in both orientations, acting as an acceptor for I⋯O halogen bonds from two TFDIB mol­ecules (I⋯O ≃ 2.80–2.90 Å). In orientation **A**, the S—CH_3_ bond vectors point approximately perpendicular to the planes of two TFDIB mol­ecules. In orientation **B**, the S—CH_3_ bonds lie closer to parallel to the TFDIB planes (Fig. 6 ▸). Thus, the DMSO mol­ecules are ‘anchored’ by the I⋯O halogen bonds, but the S—CH_3_ bond vectors can point either perpendicular or parallel to the neighbouring TFDIB rings. In the structure reported by Britton (2003 ▸), the refined site occupancies for **A** and **B** were 0.620 (17) and 0.380 (17), respectively.

On cooling of form I to 220 K, the unit cell and positions of the TFDIB mol­ecules remain com­parable to those at 297 K and both DMSO orientations **A** and **B** remain present. However, new peaks arise in the electron density corresponding to further orientations of the DMSO mol­ecules. At first it was difficult to unravel this disorder, but the situation became clear after the structure was determined at 120 K. The disorder at 220 K corresponds to *four* DMSO orientations, com­prising **A**, **B** and two new (symmetry-related) orientations described below for the 120 K structure. A significant feature of the form I structure at 220 K is that its unit-cell parameters and TFDIB positions remain com­parable to those at 297 K (Britton, 2003 ▸). Thus, cooling of the structure from 297 to 220 K causes some re-orientation of the DMSO mol­ecules, but the crystal does not yet appear to have undergone any phase transformation.

**Figure 7:**
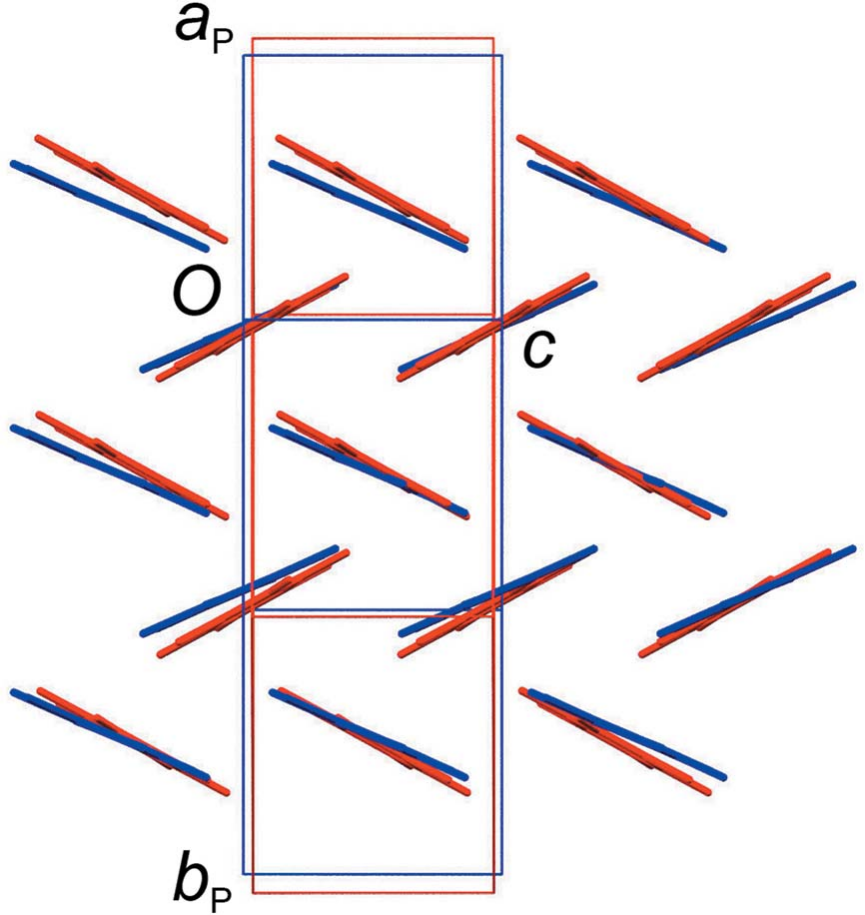
Overlay of a single layer of TFDIB mol­ecules [projecting approximately onto the (110) planes] in the form I structure at 297 K (red; Britton, 2003 ▸) and 120 K (blue). The change in the unit-cell parameters is clear, but the positions of the TFDIB mol­ecules change only very slightly.

**Figure 8:**
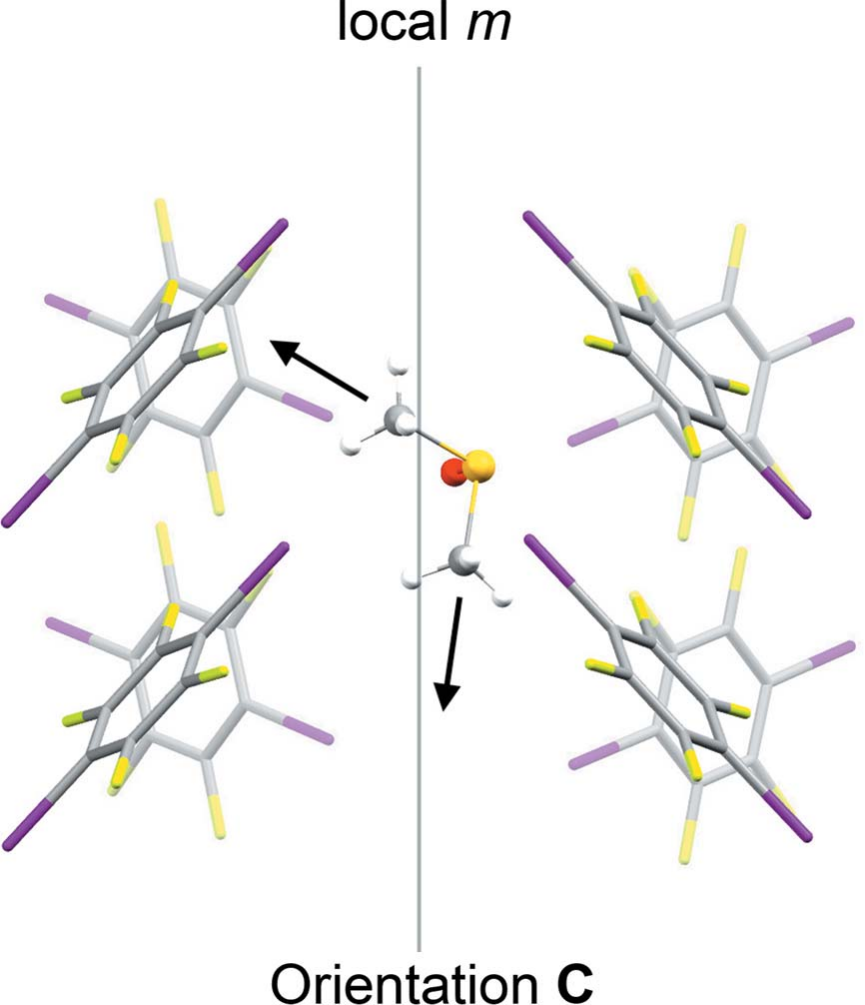
Orientation **C** for the DMSO mol­ecule in the form I structure at 120 K. The arrows indicate the directions of the S—CH_3_ bond vectors: one is equivalent to orientation **A** (Fig. 6 ▸), while one is distinct, pointing between TFDIP mol­ecules. Due to the local mirror symmetry, two locally equivalent orientations are possible for the DMSO mol­ecule, pointing either to the left or to the right in the diagram; these orientations alternate for neighbouring mol­ecules along the *c* axis (Fig. 4 ▸).

After cooling the crystal slowly (*ca* 1 K min^−1^) to 120 K, the structure changes clearly from the 297 and 220 K structures. The unit cell expands by *ca* 0.5 Å along the *c* axis and contracts by *ca* 1.0 Å along the *a* axis. Looking side-on to the TFDIB mol­ecules in one layer shows only a very subtle change com­pared to the 297 K structure (Fig. 7 ▸). However, the DMSO mol­ecules are ordered and the space-group symmetry is reduced to *P*2_1_2_1_2_1_. The DMSO orientation (labelled **C**, Fig. 8 ▸) retains essentially the same O-atom position, anchored by the I⋯O halogen bonds [I1⋯O1^i^ = 2.874 (7) Å and I4⋯O1^ii^ = 2.871 (7) Å; symmetry codes: (i) *x*, *y*, *z* − 1; (ii) −*x*, *y* − 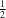, −*z* + 1]. Compared to orientations **A** and **B** (Fig. 6 ▸), the mol­ecule rotates approximately around its S—O bond. One of the S—CH_3_ bond vectors retains a position com­parable to orientation **A**, with a ‘perpendicular’ approach to the face of the neighbouring TFDIB mol­ecule. The other adopts a new position pointing approximately along the *c* axis, between TFDIB mol­ecules. Although the symmetry of the structure is clearly reduced to *P*2_1_2_1_2_1_, the TFDIB mol­ecules retain the effective mirror symmetry of the *Pnma* structure, so that two locally equivalent DMSO orientations can be envisaged, with the S—CH_3_ bond pointing towards the face of either TFDIB mol­ecule related by the local mirror symmetry (Fig. 8 ▸). Neighbouring DMSO mol­ecules along the *a* axis alternate in this respect (visible in Fig. 4 ▸), and the two additional com­ponents seen in the 220 K structure correspond to an overlay of these two orientations. It appears from the partial observation of orientation **C** at 220 K that some degree of DMSO reorientation can be tolerated within the ‘high-temperature’ TFDIB framework in form I, but the DMSO re-orientation ultimately drives the phase transformation to the ordered ‘low-temperature’ structure.

**Figure 9:**
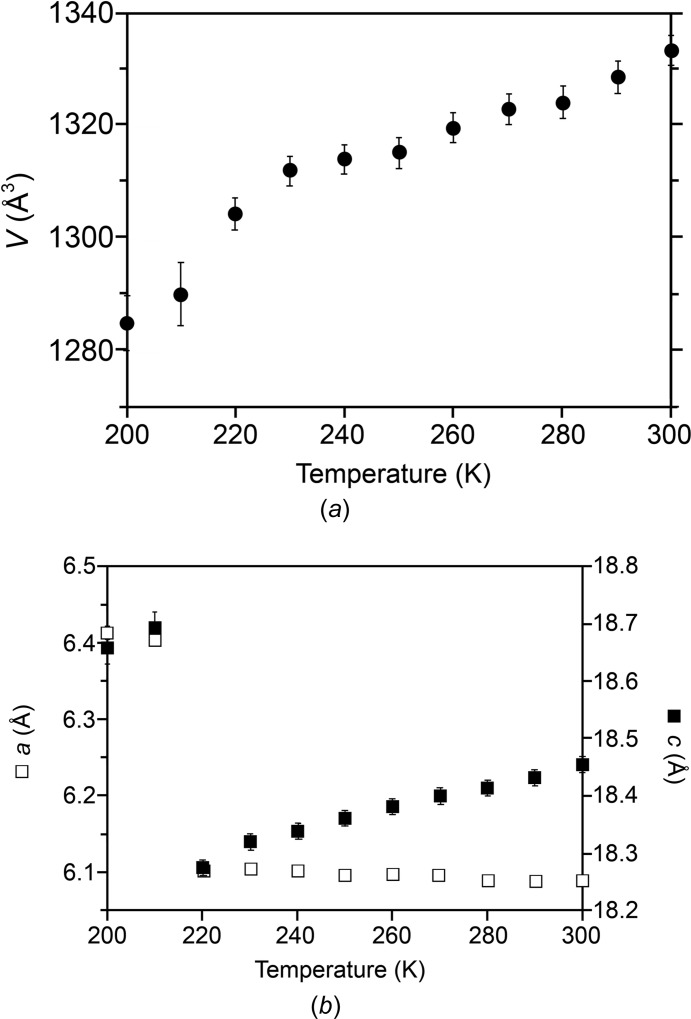
Variation in (*a*) the unit-cell volume (•) and (*b*) the *a* (□) and *c* (filled □) axis lengths over the temperature range 300→200 K for form I. Error bars (where visible) are drawn at ±(3 × s.u.). The clear discontinuities in the axis lengths correspond to the phase transformation from the ‘high-temperature’ TFDIB framework to the ‘low-temperature’ framework.

Additional temperature-dependent measurements were made to examine the unit-cell parameters in the region of the phase transformation. A crystal of form I was cooled from 300 to 200 K at a rate of *ca* 2 K min^−1^, with the unit cell determined at 10 K inter­vals. The unit-cell volume (Fig. 9 ▸ *a*) shows an approximately linear decrease over the range 300→230 K, but a clear change of gradient occurs between 230 and 220 K, suggesting that reorientation of the DMSO mol­ecules begins to take place significantly around this temperature. Clear discontinuities are evident for both the *a* and the *c* axes (Fig. 9 ▸ *b*) between the measurements made at 220 K (resembling the 297 K structure) and 210 (resembling the 120 K structure), suggesting that the reorientation is largely com­plete by 210 K. Hence, the disordered structure at 220 K captures the (average) structure of the crystal mid-transformation.

### DFT-D and *PIXEL* calculations   

To investigate the energetics of the associated inter­molecular inter­actions, models were constructed containing the various DMSO com­ponents and optimized using dispersion-corrected DFT calculations (DFT-D; see *Experimental*, §2). The purpose of the DFT-D step is to produce a model with an optimized representation of the disordered solvent mol­ecules, where the geometry from the X-ray refinement is likely to be less well defined. The pairwise inter­actions in the optimized structures were analysed using the *PIXEL* approach (Gavezzotti, 2002 ▸, 2003 ▸, 2011 ▸). The principal inter­est is the total inter­action energy between the DMSO mol­ecules and its neighbours for orientations **A**, **B** and **C** in the form I structure. In each structure, the pairwise inter­actions sorted by centroid–centroid distance show a clear set of eight DMSO–TFDIB inter­actions, as indicated in Figs. 6 ▸ and 8 ▸, with no other DMSO–TFDIB inter­actions having significant inter­action energy. Similarly, each structure shows a clear set of six significant DMSO–DMSO inter­actions, which are directly com­parable between the structures. Table 2 ▸ shows the sums of the total energies for these sets of inter­actions. It is evident that the total inter­action energy between DMSO and the TFDIB framework changes little between orientations **A**, **B** and **C**. Orientation **B** appears slightly favoured over orientation **A** in the form I structure at 297 K.^1^ For orientation **C**, however, the calculations give a clear indication: orientation **C** is favoured on account of significantly more stabilizing inter­actions between the DMSO mol­ecules. In particular, the inter­action between DMSO mol­ecules along the *a* axis (Fig. 4 ▸) has a centroid–centroid distance *ca* 0.5 Å shorter than any other DMSO–DMSO inter­action and is particularly stabilizing (*E*_tot_ = −17.3 kJ mol^−1^). It appears that this inter­action drives the ordering of the DMSO mol­ecules, resulting in the *ca* 1.0 Å contraction of the *a* axis and symmetry reduction to the space group *P*2_1_2_1_2_1_. The DMSO reorientation takes place within a TFDIB framework that is clearly flexible, as evidenced by the existence of the three closely-related framework structures reported herein, and with little consequence for the total energy of the DMSO–TFDIB inter­actions.

## Conclusion   

The existence of TFDIB·DMSO form II and the variation of the form I structure as a function of temperature shows that the layered arrangement of TFDIB mol­ecules can exhibit significant flexibility in the crystalline state. This flexibility accommodates several orientations for the DMSO mol­ecules between the layers, with apparently little variation in the DMSO–TFDIB inter­action energies. The DMSO mol­ecules are consistently anchored by accepting I⋯O halogen bonds, but their orientation can vary relative to the TFDIB mol­ecules and relative to each other. The *PIXEL* calculations suggest no clear preference for orientations **A** or **B** in form I, consistent with the observed disorder in the structure at 297 K, but they show clearly why orientation **C** is preferred in the structure at 120 K. Optimization of the inter­actions between neighbouring DMSO mol­ecules locks in an ordered arrangement, which accounts for the observed changes in the unit-cell parameters and space group on cooling of form I below *ca* 220 K. The applied combination of temperature-dependent X-ray dif­fraction measurements and inter­molecular energy calculations provides a clear picture of the temperature-dependent phase transformation in this case

## Supplementary Material

Crystal structure: contains datablock(s) Form_II, Form_I_120K, Form_I_220K, global. DOI: 10.1107/S2053229620005690/sk3750sup1.cif

Structure factors: contains datablock(s) Form_II. DOI: 10.1107/S2053229620005690/sk3750Form_IIsup2.hkl

Structure factors: contains datablock(s) Form_I_120K. DOI: 10.1107/S2053229620005690/sk3750Form_I_120Ksup3.hkl

Structure factors: contains datablock(s) Form_I_220K. DOI: 10.1107/S2053229620005690/sk3750Form_I_220Ksup4.hkl

Supporting information file. DOI: 10.1107/S2053229620005690/sk3750Form_I_120Ksup5.cml

DFT-D-optimized structures. DOI: 10.1107/S2053229620005690/sk3750sup6.txt

CCDC references: 1998986, 1998985, 1998984

## Figures and Tables

**Table 1:** Experimental details For all structures: C_6_F_4_I_2_·C_2_H_6_OS, *M*_r_ = 479.99, *Z* = 4. Experiments were carried out with Mo *K*α radiation using a Nonius KappaCCD diffractometer. Absorption was corrected for by multi-scan methods (*SORTAV*; Blessing, 1995 ▸). H-atom parameters were constrained.

	**Form II**	**Form I (120 K)**	**Form I (220 K)**
Crystal data			
Crystal system, space group	Orthorhombic, *P* *n* *m* *a*	Orthorhombic, *P*2_1_2_1_2_1_	Orthorhombic, *P* *n* *m* *a*
Temperature (K)	180	120	220
*a*, *b*, *c* (Å)	12.8308 (6), 21.3307 (12), 4.6463 (2)	10.6731 (2), 18.0023 (5), 6.5470 (2)	11.6799 (4), 18.2664 (8), 6.0984 (2)
*V* (Å^3^)	1271.65 (11)	1257.94 (5)	1301.09 (8)
μ (mm^−1^)	5.14	5.19	5.02
Crystal size (mm)	0.14 × 0.14 × 0.14	0.12 × 0.10 × 0.10	0.12 × 0.10 × 0.10

Data collection			
*T*_min_, *T*_max_	0.411, 0.464	0.475, 0.599	0.541, 0.611
No. of measured, independent and observed [*I* > 2σ(*I*)] reflections	7999, 1463, 952	11512, 2834, 2141	8062, 1520, 948
*R*_int_	0.049	0.089	0.057
(sin θ/λ)_max_ (Å^−1^)	0.649	0.649	0.650

Refinement			
*R*[*F* ^2^ > 2σ(*F* ^2^)], *wR*(*F* ^2^), *S*	0.033, 0.086, 1.01	0.043, 0.078, 0.99	0.038, 0.082, 1.06
No. of reflections	1463	2834	1520
No. of parameters	83	148	112
No. of restraints	0	0	71
Δρ_max_, Δρ_min_ (e Å^−3^)	1.01, −1.27	0.91, −1.18	0.62, −0.69
Absolute structure	–	Refined as an inversion twin.	–
Absolute structure parameter	–	0.19 (5)	–

Computer programs: *COLLECT* (Nonius, 1998 ▸), *HKL* *SCALEPACK* (Otwinowski & Minor, 1997 ▸), *HKL* *DENZO* (Otwinowski & Minor, 1997 ▸), *SHELXT* (Sheldrick, 2015*a* ▸), *SHELXL2018* (Sheldrick, 2015*b* ▸) and *Mercury* (Macrae *et al.*, 2020 ▸).

**Table 2:** Total inter­molecular inter­action energies (kJ mol^−1^) involving the DMSO mol­ecules in form I, calculated using the *PIXEL* method, applied to the DFT-D-optimized structures

Structure	Disorder com­ponent	Refinement temperature (K)	*a* (Å)	*b* (Å)	*c* (Å)	*E*_tot_ DMSO–TFDIB	*E*_tot_ DMSO–DMSO
Form I	**A**	297	11.819	18.418	6.075	−107.4	−25.0
Form I	**B**	297	11.819	18.418	6.075	−112.0	−27.4
Form I	**C**	120	10.673	18.002	6.547	−109.8	−44.6

## Footnotes

1 The fact that this is not immediately consistent with the site occupancies reported by Britton (2003 ▸) might be attributed to several factors: (i) the accuracy of the calculations; (ii) the calculations are static and do not consider entropic contributions that must become relevant at real temperatures; (iii) the DMSO orientations may be established during crystal growth, and not solely determined by their relative inter­action energies.
